# Effects of Bag Mask Ventilation and Advanced Airway Management on Adherence to Ventilation Recommendations and Chest Compression Fraction: A Prospective Randomized Simulator-Based Trial

**DOI:** 10.3390/jcm9072045

**Published:** 2020-06-29

**Authors:** Lea Vogt, Timur Sellmann, Dietmar Wetzchewald, Heidrun Schwager, Sebastian Russo, Stephan Marsch

**Affiliations:** 1 Department of Anaesthesiology, Bethesda Hospital, 47053 Duisburg, Germany; lea.vogt@posteo.de (L.V.); t.sellmann@bethesda.de (T.S.); 2 Department of Anaesthesiology 1, Witten/Herdecke University, 58455 Witten, Germany; sebastian.russo@sbk-vs.de; 3 Institution for Emergency Medicine, 59755 Arnsberg, Germany; wetzchewald@aim-arnsberg.de (D.W.); h.schwager@cardio-tours.de (H.S.); 4 Department of Intensive Care, University Hospital, 4031 Basel, Switzerland

**Keywords:** cardiopulmonary resuscitation, randomized controlled trial, bag-mask ventilation, advanced airway management, simulation

## Abstract

The role of advanced airway management (AAM) in cardiopulmonary resuscitation (CPR) is currently debated as observational studies reported better outcomes after bag-mask ventilation (BMV), and the only prospective randomized trial was inconclusive. Adherence to CPR guidelines ventilation recommendations is unknown and difficult to assess in clinical trials. This study compared AAM and BMV with regard to adherence to ventilation recommendations and chest compression fractions in simulated cardiac arrests. A total of 154 teams of 3–4 physicians were randomized to perform CPR with resuscitation equipment restricting airway management to BMV only or equipment allowing for all forms of AAM. BMV teams ventilated 6 ± 6/min and AAM teams 19 ± 8/min (range 3–42/min; *p* < 0.0001 vs. BMV). 68/78 BMV teams and 23/71 AAM teams adhered to the ventilation recommendations (*p* < 0.0001). BMV teams had lower compression fractions than AAM teams (78 ± 7% vs. 86 ± 6%, *p* < 0.0001) resulting entirely from higher no-flow times for ventilation (9 ± 4% vs. 3 ± 3 %; *p* < 0.0001). Compared to BMV, AAM leads to significant hyperventilation and lower adherence to ventilation recommendations but favourable compression fractions. The cumulative effect of deviations from ventilation recommendations has the potential to blur findings in clinical trials.

## 1. Introduction

Guidelines of cardiopulmonary resuscitation (CPR) recommend that advanced cardiac life support providers should give artificial ventilation as soon as possible [1]. Means of ventilation include mouth-to-mouth ventilation, bag-mask ventilation (BMV) and advanced airway management (AAM) with endotracheal intubation (ETI) or supraglottic airway devices (SAD). ETI protects the airway against aspiration and minimizes gastric inflation with air [2]. However, in response to potentially fatal complications of ETI [2,3]. SADs are increasingly used during CPR. Once successfully placed, both ETI and SAD allow effective ventilation without the need to interrupt chest compressions.

There are no data from randomized controlled trials supporting or refuting the routine use of any specific approach to ventilation and the timing thereof during CPR [4,5,6,7,8]. Particularly, the role of AAM in CPR remains unresolved: The only prospective randomized trial so far comparing BMV and AAM was inconclusive regarding outcome [6] while several large observational and registry studies demonstrated better outcomes using BMV [9,10,11,12,13]. However, these observational findings may relate to substantial bias as patients with a less favourable course of resuscitation may be more prone to receive AAM [5,14]. Likewise, data on the effects of AAM and BMV on no-flow times are contradictory as AAM was reported to result in higher [15,16] or unchanged [17] overall chest compression fractions than BMV. Potential mechanisms for worse CPR outcomes, if any, associated with AAM include undue interruptions of chest compressions [18,19], high failure rates (ETI), and hyperventilation and hyperinflation (ETI and SGA) [20]. 

Adherence to CPR algorithms is not always optimal and may vary between different components of the algorithm [21,22,23,24,25]. Although poor adherence to recommendations concerning airway management [26] may significantly contribute to the contradictory and inconclusive data on optimal ventilation during CPR, there are only limited data available. Airway management and its shortcomings are difficult to capture in real cases and especially so in the early phase of arrests as providing functional recording equipment or trained observers prior to the start of resuscitation efforts poses significant practical and ethical constraints. By contrast, simulation allows the recording and analysing of high-quality data under realistic conditions in a standardized manner without endangering patients [22,23]. Accordingly, the present prospective randomized study was designed to compare AAM with BMV in simulated cardiac arrests and test the hypotheses that (1) AAM results in lower adherence to ventilation recommendations and (2) in higher chest compression fractions.

## 2. Methods

### 2.1. Participants

The Working Group on Intensive Care Medicine, Arnsberg, Germany, regularly provides educational courses for physicians from all over Germany and German-speaking countries working in intensive and emergency care. Most participants are residents in their 2nd to 3rd year of postgraduate medical education in internal medicine, anaesthesia or surgery that are regularly confronted as first responders with CPR and have been exposed to medical simulations during their previous medical education to a variable extent. All participants of our educational courses were offered the opportunity to take part in voluntary simulator-based CPR workshops and informed that they would work in teams, simulations would be videotaped for scientific reasons, and video-recordings would be analysed in a strictly anonymous way. Physicians refusing to be filmed were offered identical CPR workshops without videotaping and were not included in the present study. The study was conducted in accordance with the Declaration of Helsinki, and the protocol was approved by the Ethics Committee of Ärztekammer Westfalen-Lippe (2014-657-f-N) that waived the obligation to obtain consent. The study is registered at the German Clinical Trial Registry (DRKS00022132) and reported herein according to the extensions to the CONSORT statements of the Reporting Guidelines for Health Care Simulation Research [27].

### 2.2. Study Design

In this prospective randomized single-blind trial participants from single workshops were randomly assigned to teams of three to four. Teams were then randomly allocated to perform CPR under two different conditions: (1) equipment for airway management available (AAM group) or, (2) only bag and mask available (BMV group). Medical equipment provided to all teams consisted of a manual defibrillator and an aluminium case (Ulmer Case) used for transport of emergency and resuscitation material by many German emergency systems. For AAM teams, the usual content of the case remained unchanged and included, among others, a facemask, a self-expandable bag with reservoir, supraglottic airways, endotracheal tubes, and a laryngoscope. For BMV teams, all equipment allowing advanced airway management was removed from the case so that only a facemask and a self-expandable bag with reservoir were available. Prior to the start of the scenario, BMV teams were informed that their case lacked equipment for advanced airway management due to partial restocking in the aftermath of multiple resuscitations. Except for this study-specific difference in the content of the emergency case, both versions of the simulation were identical.

### 2.3. Scenario

The full body manikin Ambu Man^®^ Wireless Mega Code Trainer (Ambu GmbH, Bad Nauheim, Germany) was used. All participants received a standardized introduction to the workshop and were made familiar with the manikin. Team members were then informed that their role during the following scenario was that of an in-hospital resuscitation team summoned to an unwitnessed cardiac arrest in their hospital’s cafeteria. The victim of the arrest (simulator) was pulseless, apnoeic, and did not react to verbal commands or painful stimuli. When attached, ventricular fibrillation could be diagnosed on the display of the defibrillator. The study period started with the first touch of the patient by a team member and ended with the return of spontaneous circulation (ROSC) upon the third defibrillation after the third cycle. Trained tutors who were instructed to refrain from any intervention until the end of the study period operated the manikins. After the simulation, tutors gave educational feedback to the teams.

### 2.4. Data Analysis

Team performance was analysed using video recordings obtained during simulations by one of the authors (L.V.). Any uncertainties in ratings were solved by jointly watching the videos with a further author (T.S. or S.M.) The first touch of the patient was defined to be the starting point for the timing of all events. Advanced airway management was defined as insertion of an ETI or a SAD. The 2015 American Heart Association (AHA) and European Resuscitation Council (ERC) guidelines for basic and advanced cardiac life support recommend for BMV the delivery of two breaths during pauses in chest compressions with an inspiratory inflation time of 1 s for each breath [1,28,29,30]. The 2015 guidelines for basic life support allow a maximum interruption in chest compressions to give two breaths of 10 s [28,30] while 2015 guidelines for advanced cardiac life support do not specify a maximum time interval and the 2010 AHA guideline for advanced cardiac life support advocates a brief pause of about 3 to 4 s [31]. Assuming equal inspiratory and expiratory time intervals, the 2015 recommendation of an inflation time of 1 s results in a pause of approximately 4 s. Accordingly, we defined a pause of ≤4 s as primary outcome strictly compatible with current ventilation recommendation and additionally present results for this target within a range of 20% (≤5 s) and 50% (≤6 s) respectively.

The 2015 AHA and ERC guidelines recommend that after insertion of an advanced airway continuous chest compressions should be performed while ventilation rates should be 10/min [29] and “approximately 10/min” [1], respectively. Accordingly, we defined an average respiration rate between 9 and 11/min as primary outcome strictly compatible with current ventilation recommendations and additionally present results for this target within a range of 20% (8–12/min)) and 50% (5–15/min), respectively. 

For each second the presence or absence of chest compressions was noted and coded if absent chest compressions (i.e., no-flow time) were related or unrelated to ventilation and/or airway management. Chest compression fraction was defined as the sum of all time intervals with chest compressions expressed as percentage of the total time interval available for chest compressions.

### 2.5. Statistics

Primary endpoints were chest compression fraction and adherence to guidelines with regard to ventilation recommendations. A difference of ≥5% between the groups for the primary outcome was considered to be of clinical significance. A power analysis, based on data of pilot experiments, revealed that a minimum of 75 teams had to be studied in each group to detect this difference with significance levels of 0.05 and 80% power. For organisational reasons, the number of available videotapes of sufficient quality could be assessed only after completion of each educational course. Secondary outcomes included request, timing, time consumption, and interruption of chest compressions for AAM. All data were analysed on an intention-to-treat basis. Data are medians (interquartile range (IQR)) unless otherwise stated. Statistical analysis was performed using IBM SPSS Statistics for Windows, version 22 (IBM Corp., Armonk, NY, USA). Student’s t-test, Mann–Whitney test, and Fisher’s exact test were applied as appropriate. A *p* < 0.05 was considered to represent statistical significance.

## 3. Results

### 3.1. Participants

**Figure 1:**
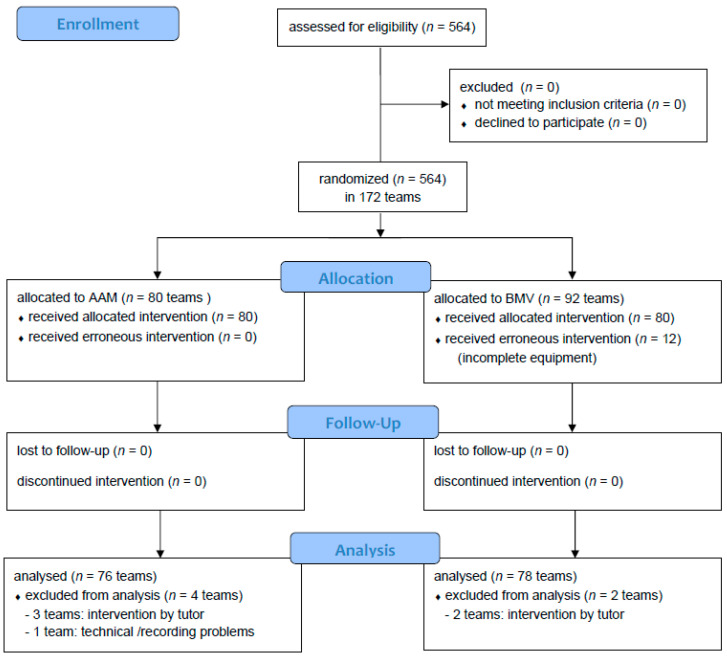
CONSORT Flow Diagram of the trial. BMV = bag-mask ventilation; AAM = advanced airway management.

Figure 1 shows the CONSORT flow chart. A total of 154 teams (76 AAM teams and 78 BMV teams) were included in the analysis.

All BMV teams performed bag-mask ventilation only. By the end of the 3rd cycle, 71/76 (93%) AAM teams had inserted an advanced airway and therefrom ETI in 19/76 and a SAD in 52/76 teams. 5/76 AAM teams had not inserted an advanced airway by the end of the 3rd cycle (no attempt in three; abandoned after failed attempt(s) in two). 17/76 AAM teams performed AAM right from the start of CPR (3 endotracheal tube, 14 supraglottic airway) while the remaining 59 AAM teams initially performed bag-valve-ventilation (58) or mouth-to-mouth ventilation (1).

### 3.2. Primary Outcomes

**Figure 2:**
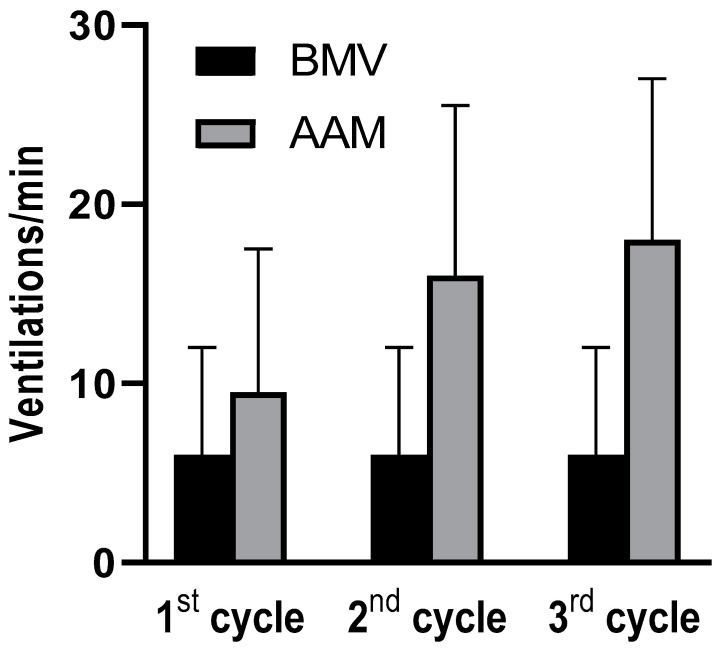
Ventilation rates during the first three cycles of CPR. Bars represent means, error bars indicate one SD. Cycles refer to blocs of chest compressions and ventilation between two defibrillations. BMV = bag-mask ventilation; AAM = advanced airway management; SD = standard deviation; CPR = cardiopulmonary resuscitation. In each cycle, ventilation rates differed significantly (*p* < 0.001) between the groups.

Over the first three cycles BMV teams ventilated at a median respiratory rate of 5/min (IQR 4–6) (range 3–24), whereas AAM teams ventilated at a respiratory rate of 17/min (IQR 14–24; range 2–37; *p* < 0.001 vs. BMV; Figure 2). Prior to insertion of an advanced airway, AAM teams ventilated at a respiratory rate of 5/min (IQR 4–7; *p* < 0.001 vs. ventilation after insertion of an advanced airway; *p* = 0.6 vs. BMV teams).

8/78 BMV teams ventilated erroneously in parallel to and without interrupting chest compressions.

The remaining 70 teams interrupted chest compressions for 2 s (IQR 2–3) for delivering two rescue breaths. 66/70, 67/70, and 69/70 teams interrupted chest compressions for ≤4 s, ≤5 s, and ≤6 s respectively. Thus, from a total of 78 BMV teams 85%, 86%, and 88% adhered to the target of interrupting chest compressions for applying rescue breaths and limiting interruptions to ≤4 s, to this target within a range of 20%, and to this target within a range of 50% respectively.

2/71 AAM teams erroneously continued ventilating in a 30:2 mode with corresponding interruptions of chest compressions after insertion of an advanced airway. In 4 of the remaining 69 AAM teams, ventilation rates were between 9 and 11/min, while in 6 and 23 teams ventilation rates were between 8–12/min and between 5–15/min, respectively. Thus, from a total of 71 AAM teams actually performing AAM, 6%, 8%, and 32% adhered to the target of uninterrupted chest compressions and a ventilation rate of approximately 10/min, to this target within a range of 20%, and to this target within a range of 50%, respectively (*p* < 0.0001 vs. adherence of corresponding values in BMV teams).

BMV teams had lower (*p* < 0.001) chest compression fractions than AAM teams: 79% (IQR 75–82) vs. 88% (IQR 83–90). This was entirely due to the no-flow times related to ventilation and/or airway management (9% (IQR 8–12) vs. 3% (IQR 1–6); *p* < 0.001) while there was no difference in no-flow times unrelated to ventilation and/or airway management (12% (IQR 9–14) vs. 10% (IQR 8–14); *p* = 0.16).

### 3.3. Secondary Outcomes

**Figure 3:**
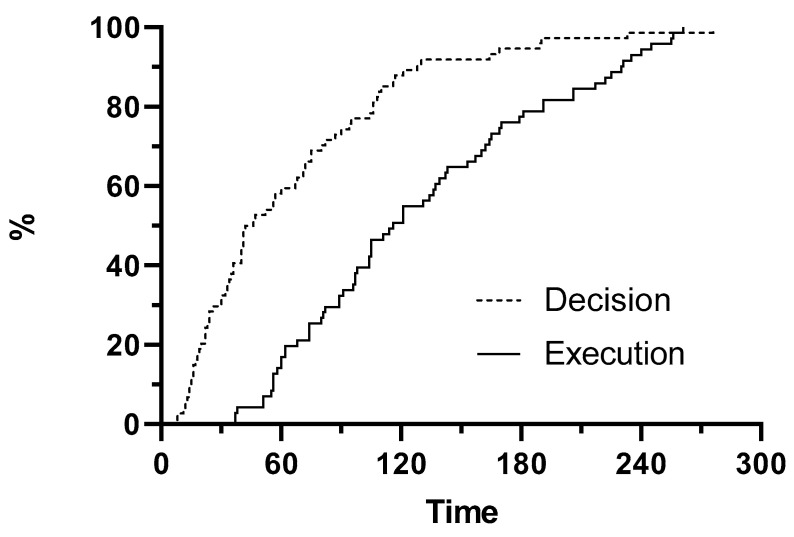
Survival curves showing the timing of decision (dotted line) and execution (solid line) of advanced airway management in AAM teams. AAM = advanced airway management.

Teams allocated to AAM took the decision to insert an advanced airway after 41 s (IQR 22–87) and completed the insertion after a further 49 s (IQR 35–94) resulting in the advanced airway being in place after 116 s (IQR 74–170) (Figure 3).

The time interval required for the placement of an advanced airway was 13 s (IQR 7–23; range 3 to 66 s) with a significant difference (*p* = 0.04) between endotracheal intubation (18 s (IQR 13–24)) and supraglottic airway (11 s (IQR 7–23)). Teams interrupted chest compressions for placing an advanced airway for 0 s (IQR 0–0: range 0 to 46; mean 2 ± 6) with no difference (*p* = 0.22) between endotracheal tube (0 s (IQR 0–5: range 0 to 46; mean 5 ± 11 s) and supraglottic airway (0 s (IQR 0–0) range 0 to 8; mean 1 ± 2 s). 56/71 AAM teams did not interrupt chest compression at all for inserting an advanced airway while 64/71 AAM teams adhered to the recommendation of a pause in chest compressions of ≤5 s, if any, for inserting an advanced airway [1]. Assessment of tube position after placement was performed by 48/71 (68%) AAM teams (43 by auscultation and 5 by auscultation and capnometry). 20/78 BMV teams asked for an advanced airway after a time interval of 109 s (IQR 72–196).

## 4. Discussion

This single-blinded randomized trial showed that compared to BMV, AAM was associated with higher than recommended ventilation rates, lower adherence to CPR guidelines’ ventilation recommendations, and more favourable chest compression fractions. 

Data on the harm or benefit of AAM during CPR are conflicting [5,6,9,10,11,12,13,15,32,33,34]. To the best of our knowledge, there is only one prospective randomized trial comparing AAM with BMV so far that, by failing to demonstrate either inferiority or non-inferiority of BMV, was formally inconclusive [6]. However, this trial reported an almost identical favourable functional survival at day 28 in the groups randomized to BMV (4.3%) and ETI (4.2%). Several large observational studies reported better CPR outcomes with BMV than with AAM [9,10,11,12,13]. These studies however may be biased to favour BMV as AAM is more likely to occur with extended duration of CPR that is strongly associated with unfavourable prognosis [5,14]. 

In addition to the hitherto undefined general role of AAM in CPR, data on optimal modality (ETI or SGA) [4,7,35] and optimal timing are inconclusive and contradictory as well. Two large observational studies found that early AAM was associated with time-dependent increased probabilities of ROSC and a functionally better survival [36,37]. However, a recent systematic review stated that the critical risk of bias for available data precluded any meaningful statement on optimal time flow of ventilation measures [5]. In the present study, we observed almost immediate AAM, as long as all required material was available.

AAM was associated with an immediate tripling of ventilation rates independently of the airway chosen. Hyperventilation is associated with persistently high airway pressures in humans [38] and decreased survival rates in an animal model [20,38]. There are, however, conflicting data on the detrimental effect of hyperventilation on coronary perfusion pressure [20,39]. 

BMV teams demonstrated a high adherence to the recommendation of limiting interrupting chest compressions for applying rescue breaths: The median interruption time of 2 s of our physician BMV providers compared favourably to a median time of 7 s of lay rescuers in a previous trial [40]. However, 6% of our teams consistently synchronized ventilation wrongly to chest compressions (asynchronous instead of synchronous ventilation in BMV; synchronous instead of asynchronous ventilation in AAM). To the best of our knowledge, there are no data on the incidence and consequences of erroneous ventilation during CPR. A median interruption time of 0 s together with a 75-percentile value of 0 s indicates that 75% of AAM teams did not interrupt chest compressions at all for the placement of an airway. However, 10% of AAM teams interrupted chest compressions more than the guideline recommendation of less than 5 s, and one third did not control the proper position of the airway inserted. 

In keeping with a previous simulator-based [16] and a large observational study [15], we observed higher chest compression fractions in AAM teams than in BMV teams. However, a recent single centre substudy of a large randomized trial reported, despite a significant reduction in no-flow time associated with ventilations, no increase in overall chest compression fraction in intubated patients compared to patients managed with BVM [17]. This observation can be explained by higher no-flow times of ETI teams associated with rhythm checks (a finding not observed in the present trial) and the use of mechanical chest compression devices. 

Strengths of this randomized trial include the large sample size, participants currently acting as first responders in real life, and identical conditions for all teams. Importantly, we were able to capture and record airway management occurring early in the course of CPR, which is extremely difficult in real cases. Moreover, restricting first responders’ airway equipment for a scientific purpose only would be difficult, if not impossible, to justify ethically and medically in patients. Limitations of simulator-based studies include the absence of real patients. However, when performance markers like chest compression fraction and technical quality of airway management are assessed, findings in simulator-based studies show a high agreement with findings in real cases [21,22,23,24,25]. Indeed, the chest compression fraction in the present study were almost identical to those reported in a large randomized trial on airway management in patients with out-of-hospital cardiac arrest [6]. Data in the AAM study arm relate to teams of physicians with regular exposure to CPR and may thus not necessarily extrapolate to less advanced first responders. However, data of the BMV arm may well apply to settings where victims of cardiac arrest are managed by basic life support for several minutes before AAM becomes available. 

The present study adds the following insights to the ongoing debate on optimal airway management: First, advanced airway management is associated at the same time with both potentially beneficial (less no-flow time) and potentially harmful (hyperventilation) features, which may partly explain conflicting data in clinical studies. Second, hyperventilation occurs consistently and starts immediately after insertion of an advanced airway regardless of the type used. Third, the incidence and degree of further deviations from ventilation recommendations in both BMV and AMV has the potential to blur true differences in clinical trials. Fourth, prolonged no-flow times are a very unlikely explanation for worse outcomes, if any, associated with AAM in general or differences in outcomes between different methods of AAM. Fifth, provided the equipment necessary is available, advanced airway management is carried out in the vast majority of cases in the very early phase of resuscitation (20% right from the start and over 90% within the first two minutes).

Given the potentially detrimental effects of hyperventilation, training and teaching of CPR should include an additional focus on adherence to ventilation recommendations. Such teaching should be easily implementable in current teaching frameworks. Potential first responders and leaders of CPR teams should be made aware of the frequent occurrence of hyperventilation after insertion of an advanced airway and other deviations from ventilation recommendations and of their duty to avoid, prevent, or correct these events. Interestingly, a favourable effect of a ventilation feedback device on the quality on manual ventilation has been recently reported in a simulator-based trial [41]. 

## 5. Conclusions

This simulator-based randomized trial of CPR demonstrates that AAM is associated with higher chest compression rates than BMV but more deviation from ventilation recommendations, and especially hyperventilation. The cumulative effect of deviations from ventilation recommendations in both BMV and AAM has the potential to blur findings in clinical trials. Training and teaching of CPR should include awareness of hyperventilation associated with AAM and measures to prevent it. 

## References

[1] Soar J., Nolan J.P., Bottiger B.W., Perkins G.D., Lott C., Carli P., Pellis T., Sandroni C., Skrifvars M.B., Smith G.B. (2015). European Resuscitation Council Guidelines for Resuscitation 2015: Section 3. Adult advanced life support. Resuscitation.

[2] Nolan J.P., Soar J. (2008). Airway techniques and ventilation strategies. Curr. Opin. Crit. Care.

[3] Lyon R.M., Ferris J.D., Young D.M., McKeown D.W., Oglesby A.J., Robertson C. (2010). Field intubation of cardiac arrest patients: A dying art?. Emerg. Med. J..

[4] Benoit J.L., Gerecht R.B., Steuerwald M.T., McMullan J.T. (2015). Endotracheal intubation versus supraglottic airway placement in out-of-hospital cardiac arrest: A meta-analysis. Resuscitation.

[5] Granfeldt A., Avis S.R., Nicholson T.C., Holmberg M.J., Moskowitz A., Coker A., Berg K.M., Parr M.J., Donnino M.W., Soar J. (2019). Advanced airway management during adult cardiac arrest: A systematic review. Resuscitation.

[6] Jabre P., Penaloza A., Pinero D., Duchateau F.X., Borron S.W., Javaudin F., Richard O., de Longueville D., Bouilleau G., Devaud M.L. (2018). Effect of Bag-Mask Ventilation vs Endotracheal Intubation During Cardiopulmonary Resuscitation on Neurological Outcome After Out-of-Hospital Cardiorespiratory Arrest: A Randomized Clinical Trial. JAMA.

[7] Wang H.E., Szydlo D., Stouffer J.A., Lin S., Carlson J.N., Vaillancourt C., Sears G., Verbeek R.P., Fowler R., Idris A.H. (2012). Endotracheal intubation versus supraglottic airway insertion in out-of-hospital cardiac arrest. Resuscitation.

[8] Benger J.R., Kirby K., Black S., Brett S.J., Clout M., Lazaroo M.J., Nolan J.P., Reeves B.C., Robinson M., Scott L.J. (2018). Effect of a Strategy of a Supraglottic Airway Device vs Tracheal Intubation During Out-of-Hospital Cardiac Arrest on Functional Outcome: The AIRWAYS-2 Randomized Clinical Trial. JAMA.

[9] Andersen L.W., Raymond T.T., Berg R.A., Nadkarni V.M., Grossestreuer A.V., Kurth T., Donnino M.W., for the American Heart Association’s Get With The Guidelines-Resuscitation Investigators (2016). Association Between Tracheal Intubation During Pediatric In-Hospital Cardiac Arrest and Survival. JAMA.

[10] Andersen L.W., Granfeldt A., Callaway C.W., Bradley S.M., Soar J., Nolan J.P., Kurth T., Donnino M.W., for the American Heart Association’s Get With The Guidelines-Resuscitation Investigators (2017). Association Between Tracheal Intubation During Adult In-Hospital Cardiac Arrest and Survival. JAMA.

[11] Hanif M.A., Kaji A.H., Niemann J.T. (2010). Advanced airway management does not improve outcome of out-of-hospital cardiac arrest. Acad. Emerg. Med..

[12] Hasegawa K., Hiraide A., Chang Y., Brown D.F. (2013). Association of prehospital advanced airway management with neurologic outcome and survival in patients with out-of-hospital cardiac arrest. JAMA.

[13] Sanghavi P., Jena A.B., Newhouse J.P., Zaslavsky A.M. (2015). Outcomes After Out-of-Hospital Cardiac Arrest Treated by Basic vs Advanced Life Support. JAMA Intern. Med..

[14] Andersen L.W., Grossestreuer A.V., Donnino M.W. (2018). “Resuscitation time bias”—A unique challenge for observational cardiac arrest research. Resuscitation.

[15] Yeung J., Chilwan M., Field R., Davies R., Gao F., Perkins G.D. (2014). The impact of airway management on quality of cardiopulmonary resuscitation: An observational study in patients during cardiac arrest. Resuscitation.

[16] Wiese C.H., Bartels U., Schultens A., Steffen T., Torney A., Bahr J., Graf B.M. (2008). Influence of airway management strategy on “no-flow-time” during an “Advanced life support course” for intensive care nurses - A single rescuer resuscitation manikin study. BMC Emerg. Med..

[17] Malinverni S., Bartiaux M., Cavallotto F., De Longueville D., Mols P., Gorlicki J., Adnet F. (2019). Does endotracheal intubation increases chest compression fraction in out of hospital cardiac arrest: A substudy of the CAAM trial. Resuscitation.

[18] Wang H.E., Simeone S.J., Weaver M.D., Callaway C.W. (2009). Interruptions in cardiopulmonary resuscitation from paramedic endotracheal intubation. Ann. Emerg. Med..

[19] Abo B.N., Hostler D., Wang H.E. (2007). Does the type of out-of-hospital airway interfere with other cardiopulmonary resuscitation tasks?. Resuscitation.

[20] Aufderheide T.P., Sigurdsson G., Pirrallo R.G., Yannopoulos D., McKnite S., von B.C., Sparks C.W., Conrad C.J., Provo T.A., Lurie K.G. (2004). Hyperventilation-induced hypotension during cardiopulmonary resuscitation. Circulation.

[21] Abella B.S., Alvarado J.P., Myklebust H., Edelson D.P., Barry A., O’Hearn N., Vanden Hoek T.L., Becker L.B. (2005). Quality of Cardiopulmonary Resuscitation During In-Hospital Cardiac Arrest. JAMA: J. Am. Med. Assoc..

[22] Marsch S.C., Muller C., Marquardt K., Conrad G., Tschan F., Hunziker P.R. (2004). Human factors affect the quality of cardiopulmonary resuscitation in simulated cardiac arrests. Resuscitation.

[23] Marsch S.C., Tschan F., Semmer N., Spychiger M., Breuer M., Hunziker P.R. (2005). Performance of first responders in simulated cardiac arrests. Crit. Care Med..

[24] Tschan F., Vetterli M., Semmer N.K., Hunziker S., Marsch S.C. (2011). Activities during interruptions in cardiopulmonary resuscitation: A simulator study. Resuscitation.

[25] Wik L., Kramer-Johansen J., Myklebust H., Sorebo H., Svensson L., Fellows B., Steen P.A. (2005). Quality of Cardiopulmonary Resuscitation During Out-of-Hospital Cardiac Arrest. JAMA: J. Am. Med. Assoc..

[26] Sall F.S., De L.A., Pazart L., Pugin A., Capellier G., Khoury A. (2018). To intubate or not: Ventilation is the question. A manikin-based observational study. BMJ Open Respir Res..

[27] Cheng A., Kessler D., Mackinnon R., Chang T.P., Nadkarni V.M., Hunt E.A., Duval-Arnould J., Lin Y., Cook D.A., Pusic M. (2016). Reporting Guidelines for Health Care Simulation Research: Extensions to the CONSORT and STROBE Statements. Simul. Healthc..

[28] Kleinman M.E., Brennan E.E., Goldberger Z.D., Swor R.A., Terry M., Bobrow B.J., Gazmuri R.J., Travers A.H., Rea T. (2015). Part 5: Adult Basic Life Support and Cardiopulmonary Resuscitation Quality: 2015 American Heart Association Guidelines Update for Cardiopulmonary Resuscitation and Emergency Cardiovascular Care. Circulation.

[29] Link M.S., Berkow L.C., Kudenchuk P.J., Halperin H.R., Hess E.P., Moitra V.K., Neumar R.W., O’Neil B.J., Paxton J.H., Silvers S.M. (2015). Part 7: Adult Advanced Cardiovascular Life Support: 2015 American Heart Association Guidelines Update for Cardiopulmonary Resuscitation and Emergency Cardiovascular Care. Circulation.

[30] Perkins G.D., Handley A.J., Koster R.W., Castren M., Smyth M.A., Olasveengen T., Monsieurs K.G., Raffay V., Grasner J.T., Wenzel V. (2015). European Resuscitation Council Guidelines for Resuscitation 2015: Section 2. Adult basic life support and automated external defibrillation. Resuscitation.

[31] Neumar R.W., Otto C.W., Link M.S., Kronick S.L., Shuster M., Callaway C.W., Kudenchuk P.J., Ornato J.P., McNally B., Silvers S.M. (2010). Part 8: Adult advanced cardiovascular life support: 2010 American Heart Association Guidelines for Cardiopulmonary Resuscitation and Emergency Cardiovascular Care. Circulation.

[32] Gough C.J.R., Nolan J.P. (2018). To intubate or not to intubate?. Curr. Opin. Crit. Care.

[33] Soar J., Nolan J.P. (2013). Airway management in cardiopulmonary resuscitation. Curr. Opin. Crit. Care.

[34] Lupton J.R., Schmicker R.H., Stephens S., Carlson J.N., Callaway C., Herren H., Idris A.H., Sopko G., Puyana J.C.J., Daya M.R. (2020). Outcomes With the Use of Bag-Valve-Mask Ventilation During Out-of-hospital Cardiac Arrest in the Pragmatic Airway Resuscitation Trial. Acad. Emerg. Med..

[35] Wang H.E., Schmicker R.H., Daya M.R., Stephens S.W., Idris A.H., Carlson J.N., Colella M.R., Herren H., Hansen M., Richmond N.J. (2018). Effect of a Strategy of Initial Laryngeal Tube Insertion vs Endotracheal Intubation on 72-Hour Survival in Adults With Out-of-Hospital Cardiac Arrest: A Randomized Clinical Trial. JAMA.

[36] Benoit J.L., McMullan J.T., Wang H.E., Xie C., Xu P., Hart K.W., Stolz U., Lindsell C.J. (2019). Timing of Advanced Airway Placement after Witnessed Out-of-Hospital Cardiac Arrest. Prehosp. Emerg. Care.

[37] Izawa J., Iwami T., Gibo K., Okubo M., Kajino K., Kiyohara K., Nishiyama C., Nishiuchi T., Hayashi Y., Kiguchi T. (2018). Timing of advanced airway management by emergency medical services personnel following out-of-hospital cardiac arrest: A population-based cohort study. Resuscitation.

[38] O’Neill J.F., Deakin C.D. (2007). Do we hyperventilate cardiac arrest patients?. Resuscitation.

[39] Gazmuri R.J., Ayoub I.M., Radhakrishnan J., Motl J., Upadhyaya M.P. (2012). Clinically plausible hyperventilation does not exert adverse hemodynamic effects during CPR but markedly reduces end-tidal PCO(2). Resuscitation.

[40] Beesems S.G., Wijmans L., Tijssen Jan G.P., Koster R.W. (2013). Duration of Ventilations During Cardiopulmonary Resuscitation by Lay Rescuers and First Responders. Circulation.

[41] Khoury A., De Luca A., Sall F.S., Pazart L., Capellier G. (2019). Ventilation feedback device for manual ventilation in simulated respiratory arrest: A crossover manikin study. Scand. J. Trauma Resusc. Emerg. Med..

